# Progress and prospects in harnessing wild relatives for genetic enhancement of salt tolerance in rice

**DOI:** 10.3389/fpls.2023.1253726

**Published:** 2024-01-31

**Authors:** Guntupalli Padmavathi, Umakanth Bangale, K. Nagendra Rao, Divya Balakrishnan, Melekote Nagabhushan Arun, Rakesh Kumar Singh, Raman Meenakshi Sundaram

**Affiliations:** ^1^ Crop Improvement Section, Plant Breeding, ICAR-Indian Institute of Rice Research (ICAR-IIRR), Hyderabad, India; ^2^ Genetics and Plant Breeding, Sugarcane Research Station, Vuyyuru, India; ^3^ Crop Production Section, Agronomy, ICAR-Indian Institute of Rice Research (ICAR-IIRR), Hyderabad, India; ^4^ Crop Diversification and Genetics Section, International Center for Biosaline Agriculture (ICBA), Dubai, United Arab Emirates

**Keywords:** rice, salt tolerance, mechanisms, wild gene pool, traditional breeding, MAS, transgenics, genomics

## Abstract

Salt stress is the second most devastating abiotic stress after drought and limits rice production globally. Genetic enhancement of salinity tolerance is a promising and cost-effective approach to achieve yield gains in salt-affected areas. Breeding for salinity tolerance is challenging because of the genetic complexity of the response of rice plants to salt stress, as it is governed by minor genes with low heritability and high G × E interactions. The involvement of numerous physiological and biochemical factors further complicates this complexity. The intensive selection and breeding efforts targeted towards the improvement of yield in the green-revolution era inadvertently resulted in the gradual disappearance of the loci governing salinity tolerance and a significant reduction in genetic variability among cultivars. The limited utilization of genetic resources and narrow genetic base of improved cultivars have resulted in a plateau in response to salinity tolerance in modern cultivars. Wild species are an excellent genetic resource for broadening the genetic base of domesticated rice. Exploiting novel genes of underutilized wild rice relatives to restore salinity tolerance loci eliminated during domestication can result in significant genetic gain in rice cultivars. Wild species of rice, *Oryza rufipogon* and *Oryza nivara*, have been harnessed in the development of a few improved rice varieties like Jarava and Chinsura Nona 2. Furthermore, increased access to sequence information and enhanced knowledge about the genomics of salinity tolerance in wild relatives has provided an opportunity for the deployment of wild rice accessions in breeding programs, while overcoming the cross-incompatibility and linkage drag barriers witnessed in wild hybridization. Pre-breeding is another avenue for building material that are ready for utilization in breeding programs. Efforts should be directed towards systematic collection, evaluation, characterization, and deciphering salt tolerance mechanisms in wild rice introgression lines and deploying untapped novel loci to improve salinity tolerance in rice cultivars. This review highlights the potential of wild relatives of *Oryza* to enhance tolerance to salinity, track the progress of work, and provide a perspective for future research.

## 1 Introduction

Cultivated rice, which primarily includes *Oryza sativa* (Asian cultivated rice) and *Oryza glaberrima* (African cultivated rice), contains 22 wild species that are not cultivated (Solis et al., 2020). *O. sativa* is cultivated worldwide, whereas *O. glaberrima*, is predominantly grown in Africa. Wild rice ancestors have adapted to various geographically distinct habitats (Atwell et al., 2014). The 22 wild ancestors constituted the largest gene pool. *Oryza* species are highly variable and comprise 11 distinct genomes, including six diploids (AA, BB, CC, EE, FF, and GG) and five allotetraploids (BBCC, CCDD, KKLL, HHJJ, and HHKK) (Stein et al., 2018). They differ in morphological characteristics such as growth habit, plant height, flowering, leaf size, panicle size, and branching, awning, and seed size.

**Figure 1:**
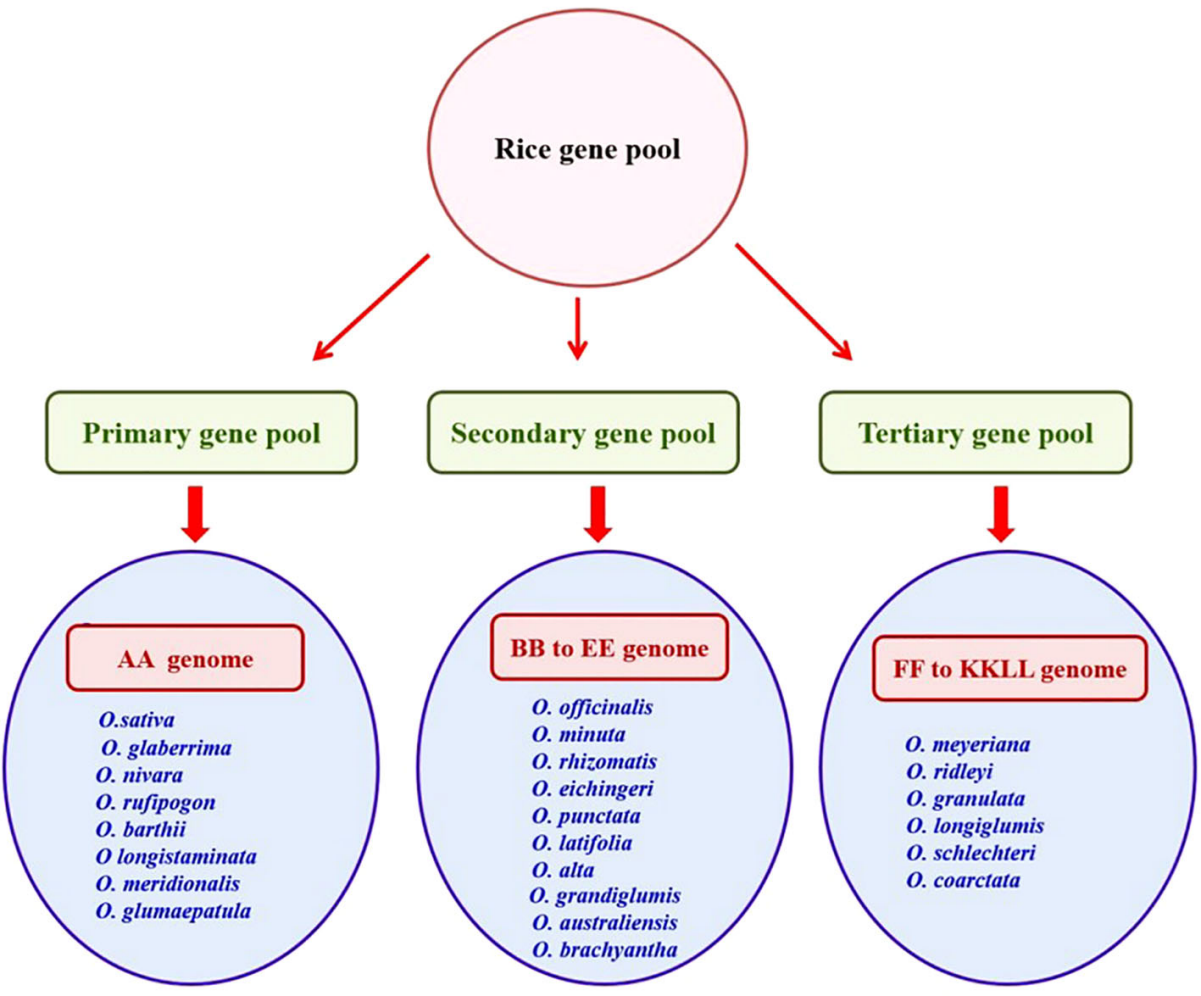
Gene pools of rice representing various wild and related species.

Wild relatives of rice are grouped into three gene pools (primary, secondary, and tertiary) based on their ease of hybridization with cultivated rice and phylogenetic relationships (Harlan and de Wet, 1971). The primary gene pool comprises the *O. sativa* complex (AA genome) with close relatives of cross-compatible rice and a secondary gene pool consisting of *O. officinalis* complex (BB to FF genomes) with less closely related species and a tertiary gene pool constituted with *O. meyeriana* complex, *O. ridleyi* complex, and *O. schlechteria* complex (GG to KKLL genomes) with more distant relatives of rice, necessitating embryo rescue, chromosome doubling, or bridging species to facilitate gene transfer (Solis et al., 2020) (**Figure 1**).

Studies have revealed that indigenous species of rice could potentially be used as genetic resources for abiotic stresses such as submergence (Luo et al., 2020; Sasayama et al., 2022) drought (Hamzelou et al., 2020; Anilkumar et al., 2023), heat (Prasanth et al., 2012; Khan et al., 2019; Jagadish et al., 2021), etc. However, few limited published studies have reported the significance of wild rice species in elevating salinity tolerance in cultivars (Solis et al., 2020).

Indeed, very few review articles have focused on the genetic enhancement of salinity tolerance in wild *O. sativa* species. Hence, in this manuscript, we have reviewed the impact of salinity effects in rice, the importance of wild rice species in breeding for salinity tolerance, characterization and mechanisms of tolerance in wild rice, deployment of wild rice genes in rice breeding, and challenges and opportunities for the incorporation of wild gene loci to develop salt-resilient rice cultivars.

## 2 Importance of wild species in rice breeding

Wild progenitors adapted to various changing climatic conditions display tremendous genetic diversity and carry genetic loci associated with unique agronomic and adaptive traits (Hellwig et al., 2021). The basic requirement of a successful crop breeding program is the availability and accessibility of genetic variability in target traits (Perrino and Perrino, 2020). When there is exhaustion in genetic variation among domesticated genotypes, breeders are left with access the diversity available in land races and wild ancestors as alternate sources of variability.

Asian cultivated rice has evolved from its wild ancestor *Oryza rufipogon* through human efforts (Wang et al., 1992). Similarly, the African rice *O. glaberrima* was domesticated from the African wild progenitor *Oryza barthii*. Erect growth, non-shattering, and increased grain number and weight were targeted during selective breeding. The intense selection and breeding that occurred during domestication could have probably resulted in the loss of genetic variability and the loci associated with stress tolerance in cultivars. *O. sativa* represents <20% of the total variation compared to that found in various wild species (Stein et al., 2018), and significant natural variability for salt tolerance still exists in wild species (Munns et al., 2016), which can be exploited to improve cultivated rice. There are certain wild species such as *Oryza coarctata*, a distantly related wild rice that thrives under extreme salinity (450 mM NaCl). However, the crossability barrier limits their utility.

Owing to tall plant stature, photosensitivity, poor grain type, low seed set, high grain shattering, and low yield potential, natural wild accessions are regarded as poor agronomic performers (Sanchez et al., 2013). Despite this, the recovery of widely adaptable cultivars to diverse challenging environments would be higher when wild relatives of rice are used in the crossing program (Jin et al., 2021). Insights into morphological and physiological responses to salt stress are crucial for exploiting stress tolerance through distant crosses. The extent to which wild rice progenitors demonstrate tolerance and the probable genetic defense strategies behind tolerance remains partially understood. Crossing barriers, linkage drag, and epistatic effects from unadapted wild genes when introgressed into elite cultivars may complicate mainstream breeding. However, advances in genomic tools and techniques, particularly embryo rescue techniques and deploying molecular marker-based advanced backcrosses, identification, selection, and incorporation of target quantitative trait loci (QTLs) into elite varieties, could successfully generate improved versions of cultivars (Dai et al., 2022).

The genetic wealth of rice diversity was systematically preserved in gene banks. As of December 2023 (URL-https://www.irri.org/international-rice-genebank), the International Rice Gene Bank Collection Information System (IRGCIS) of the International Rice Research Institute (IRRI) documented 1,32,000 accessions of both cultivated and wild rice. Nevertheless, only a few wild accessions have been evaluated using various salt-screening methods (Kumar et al., 2015). The primary gene pool consisting of cultivated rice species (*O. sativa* and *O. glaberrima*) and wild rice species (*Oryza nivara*, *O. rufipogon*, *O. barthii*, *Oryza longistaminata*, *Oryza meridionalis*, and *Oryza glumaepatula*) are analyzed for detecting tolerant wild accessions in limited research investigations. A large untapped genetic diversity is available in the secondary and tertiary gene pools, which are additional sources of new salinity tolerance genes. These gene pools can be used to introduce beneficial genes into superior rice varieties (Prusty et al., 2018; Solis et al., 2020).

Over the past three decades, there has been substantial advancement in the genetic diversity analysis of wild rice resources and the selection of suitable donor parents. Following the standard Yoshida solution culture method (Gregorio et al., 1997) and the modified Yoshida solution culture method (Singh et al., 2010), several germplasm accessions were screened at the seedling stage to identify the donors. A few accessions of *O. rufipogon* and *O. nivara* that survived salinity levels as high as 12 dSm^−1^ (Habiba et al., 2015) were identified. *O. rufipogon* is considered the best donor among the closest wild relatives belonging to the AA genome, followed by the distant wild relatives *O. coarctata*, *O. latifolia*, and *Oryza alta* (Solis et al., 2020).

At the ICAR-National Institute of Plant Biotechnology, India, 800 accessions of *O. nivara* and *O. rufipogon* collected across ecologies were evaluated (Singh et al., 2018). The accession NKSWR 173 recorded high seedling stage salt tolerance (150 mM NaCl) (Mishra et al., 2016). A survey of 22 accessions of wild species screened under high salinity (240 mM NaCl) in a hydroponic system revealed seven accessions, *Oryza minuta*, *Oryza grandiglumis*, *Oryza latifolia*, *O. alta*, *Oryza rhizomatis*, *O. coarctata*, and *Oryza eichingeri*, with higher levels of tissue tolerance and chlorophyll preservation in leaves as compared to the donor parents for salinity tolerance(Pokkali, Nona Bokra, and FL478) and salt-sensitive checks (IR29 and IR75862-206-2-8-3) (Prusty et al., 2018). Mondal et al. (2018) and Mishra et al. (2022) studied the halophytic wild species *O. coarctata* abundant in Indian coastal regions as a source of salinity tolerance genes.

In the past two decades, genome sequencing technology has improved the utilization of genetic variation in wild *Oryza* species for crop improvement. Efforts to sequence wild rice genomes began in 2003 with the establishment of the International Oryza Map Alignment Project (IOMAP). It has provided an in-depth characterization of wild rice genomes to discover and exploit genes/genomic regions governing diverse traits for their transfer into cultivated rice (Wing et al., 2005). Stein et al. (2018) sequenced genomes of seven wild species (*O. rufipogon*, *O. nivara*, *O. barthii*, *O. glumaepatula*, *O. meridionalis*, *O. punctata*, and *L. perrieri*) and deposited at National Center for Biotechnology Information (NCBI), Bethesda, Maryland and provided an array of diversity panels. The genome of *O. coarctata* has been sequenced at ICAR-NIPB, India, which would be a valuable addition to the I-OMAP project and would supplement the current genomic resources of wild and cultivated species (Mondal et al., 2018). This provides insight into the genetic makeup of *O. coarctata* and broadens the gene pool for the enhancement of cultivated rice. Multiple studies have employed whole genome sequencing and resequencing information from cultivated and wild rice population species to identify genomic regions harboring improved agronomic traits as well as adaptive traits, such as resistance to biotic stresses and tolerance to abiotic stresses, including salinity tolerance (Huang et al., 2012; Xu et al., 2012).

## 3 Impact of salt stress on rice

Rice is the main nutritional source for more than half of the world’s population (Zhou et al., 2020). Globally, rice is the third-largest cereal grown in 162 million hectares, with an annual production of 755 million tons (FAO, 2022). Soil salinity and alkalinity are the leading abiotic stressors in coastal and inland areas, followed by drought (Islam et al., 2021). Even moderate salt stress has been reported to reduce the rice yield by 68% (Naheed et al., 2007). The changing global climate is expected to cause a significant increase in soil salinization owing to inadequate rainfall, high evaporation rates, and seawater intrusion (Cheng et al., 2020).

Salt-affected soils are broadly classified as sodic–alkaline, saline, or saline–sodic soils (Eynard et al., 2005). In alkaline soils, carbonates and bicarbonates of sodium and magnesium are the most prevalent anions, whereas chlorides and sulfates of sodium and magnesium are frequently found in saline soils. Sodic soils have pH >8.5, the electrical conductivity of saturation extract (ECe) <4 dS m^−1^ and exchangeable sodium percentage (ESP) >15. Saline soils near the coastal regions, ECe >4 dS m^−1^, pH < 8.5, and ESP <15. Saline sodic soils exhibit saline and sodic characteristics, such as variable pH, ECe ≥4 dS m^−1^, and ESP ≥15%.

**Figure 2:**
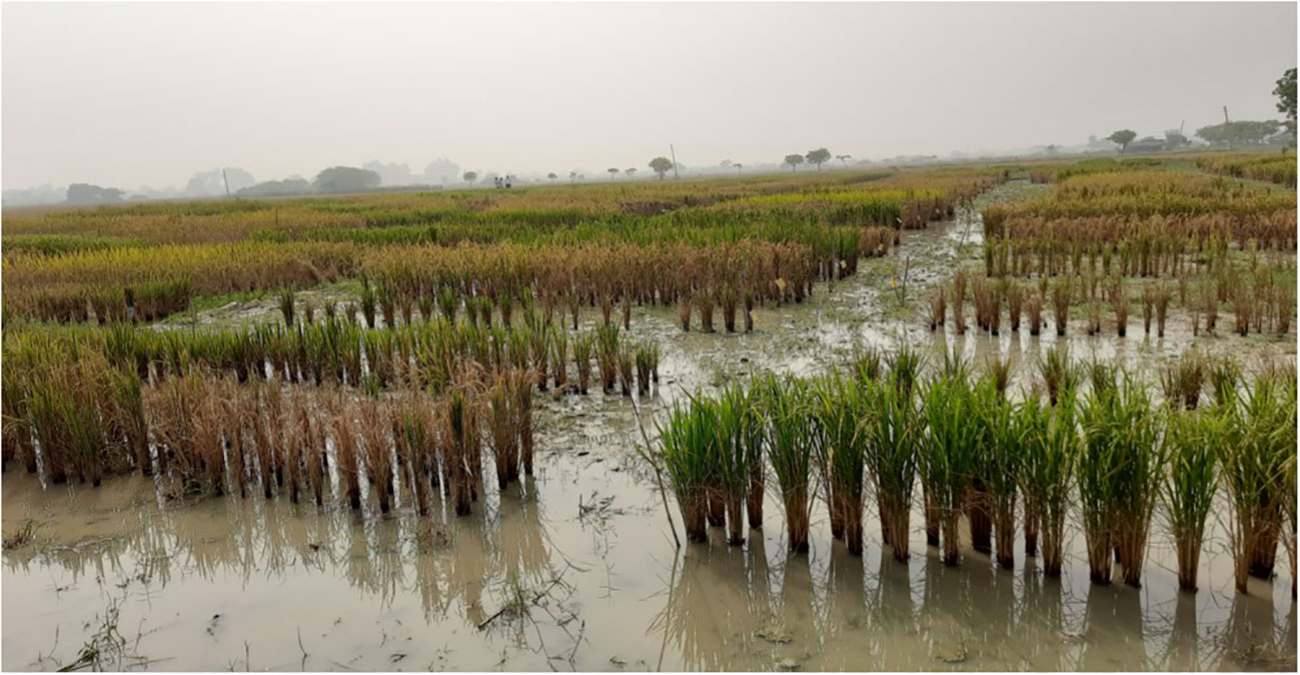
Rice genotypes showing field symptoms of alkalinity stress grown at Chandra Shekhar Azad University of Agriculture & Technology (CSAUAT), Kanpur, India.

Plants are categorized into halophytes and glycophytes based on their responses to salt stress, (Flowers and Colmer, 2008; Mishra and Tanna, 2017). Halophytes thrive in highly saline soils. Most of the crops are salt-sensitive glycophytes. Rice has a threshold salt concentration of >30 mM NaCl (ECe = 3 dS m^−1^) (USDA et al., 2008). A decrease in yield of 12% was reported for each unit, exceeding the specified threshold (Reddy et al., 2014). Excessive salinity causes osmotic stress and ion toxicity in crop plants. Osmotic pressure reduces soil osmotic potential and causes decreased water uptake, further inhibiting stomatal opening, photosynthesis, elongation, and cell proliferation. This results in a slower growth. Ionic stress causes rapid accumulation of toxic Na^+^ and Cl^−^, disrupting metabolic processes, resulting in early senescence and reduced stomatal conductance, leading to decreased photosynthesis, biomass, and poor yield (Yu et al., 2017). Salinity stress, apart from reducing germination, causes whitening of affected leaf tips, leaf rolling, stunted plant growth, patchy appearance in the field, reduced tillering, delayed panicle emergence, length and the number of panicles, reduces pollen viability, spikelet fertility, spikelet number/panicle, and ultimately grain yield, and in cases of increased severity, results in the death of the rice plant (**Figure 2**).

Plants adapt to high salt through various physiological and biochemical defense systems namely high initial seedling vigor, early maturity, sodium exclusion, sodium compartmentalization in vacuoles of roots and older leaves, osmotic adjustment, control of Reactive Oxygen Species (ROS) through production of antioxidants, and programmed cell death.

During the domestication of cultivated rice from local landraces and traditional varieties, selection pressure for productivity traits favored a few genes at the expense of many others. Hence, domesticated varieties have less genetic diversity for other non-selected traits, such as biotic and abiotic stresses, including salinity tolerance, compared to wild species and landraces. This implies that the cultivated gene pool has a narrow genetic base; hence, further gain in salinity tolerance is difficult to achieve. Consequently, the developed cultivars were found to be salt sensitive or moderately tolerant to salinity. Interestingly, wild rice gene pools possess extensive genetic diversity as they grow in undisturbed natural habitats. This diversity can enrich the cultivated gene pool with higher salt tolerance through introgression of wild genes. Salt stress profoundly affects various morphological, physiological, and biochemical processes in rice plants (**Table 1**).

**Table 1:** Effects of salt stress on morphological, physiological, and biochemical characteristics in rice.

Effects	References
1 Morphological characteristics	
Reduced seed germination and leaf expansion	Ghosh et al. (2016)
Impeding overall plant growth	Quan et al. (2018)
Decreased leaf area, length of roots and shoots, fresh and dry weights of biomass	Barua et al. (2015); Hussain et al. (2017); Dugasa et al. (2019)
Reduced number of tillers, panicles, spikelets per panicle, length of panicles, spikelet fertility and 1000 grain weight	Clermont-Dauphin et al. (2010); Mojakkir et al. (2015); Razzaque et al. (2017); Negrao et al. (2017); Rodriguez-Llorente et al. (2019)
Hampered total dry matter production and leaf area	Singh et al. (2009); Channa et al. (2023)
Caused curled, brown, and dry leaves	Munns (2005)
Ageing of older leaves and premature plant mortality	Sirault et al. (2009); Amirjani (2010)
Leaf tip burning, stunted growth, necrotic lesions on old leaves and reduced survival of plants	Kumar et al. (2007); Mohammadi et al. (2013)
Reduced the average number and length of roots per plant causing poor nutrient uptake and decreased grain yield	Hussain et al. (2017)
Reduced panicle emergence, and flowering	Saade et al. (2016), Puram et al. (2017)
Delayed flowering, reduced pollen viability and seed set.	Reddy et al. (2017); Irakoze et al. (2020)
Lowered seedling growth, plant height grain number and lower yield	Banumathy et al. (2018); Soares et al. (2021)
2 Physiological characteristics	
Decreased leaf photosynthesis, respiration rate and biomass	Chinnusamy et al. (2005); Jamil et al. (2012); Quan et al. (2018); Tsai et al. (2019); Channa et al. (2021).
Hastened senescence of leaves	Munns (2002)
Minimized turgor pressure in plant tissues limiting the proliferation of both root and shoot cells	Zelm et al. (2020)
Triggered stomatal closure, impeding carbon dioxide uptake and photosynthesis	Zhao et al. (2020)
Decreased biosynthesis of leaf chlorophyll pigments	Chutipaijit et al. (2011)
Lowered harvest index.	Gholizadeh et al. (2014)
3 Biochemical characteristics	
Caused ion toxicity and cellular damage by increasing Na^+^ concentration and Cl^-^ imbalance in cytosol	Hanin et al. (2016)
Triggered biosynthesis of osmolytes (Fructose, sucrose, mannitol, glycerol, trehalose proline, glycine betaine, glutamic acid and secondary metabolites) within cells	Sami et al. (2016); Hussain et al. (2016)
Suppressed enzyme activities and impedes protein synthesis	Horie et al. (2012)
Depleted micro (Mg, Zn, and Fe) and macro (N, P, and K) nutrients interrupting normal nutrient uptake	Razzaq et al. (2020)
Enhanced Na^+^ flow into cells and lowers K^+^/Na^+^ ratio	Chen et al. (2007)
Stimulated production of ROS (Hydrogen peroxide and superoxide).	Xie et al. (2019)

## 4 Defense strategies of salt tolerance in rice

**Figure 3:**
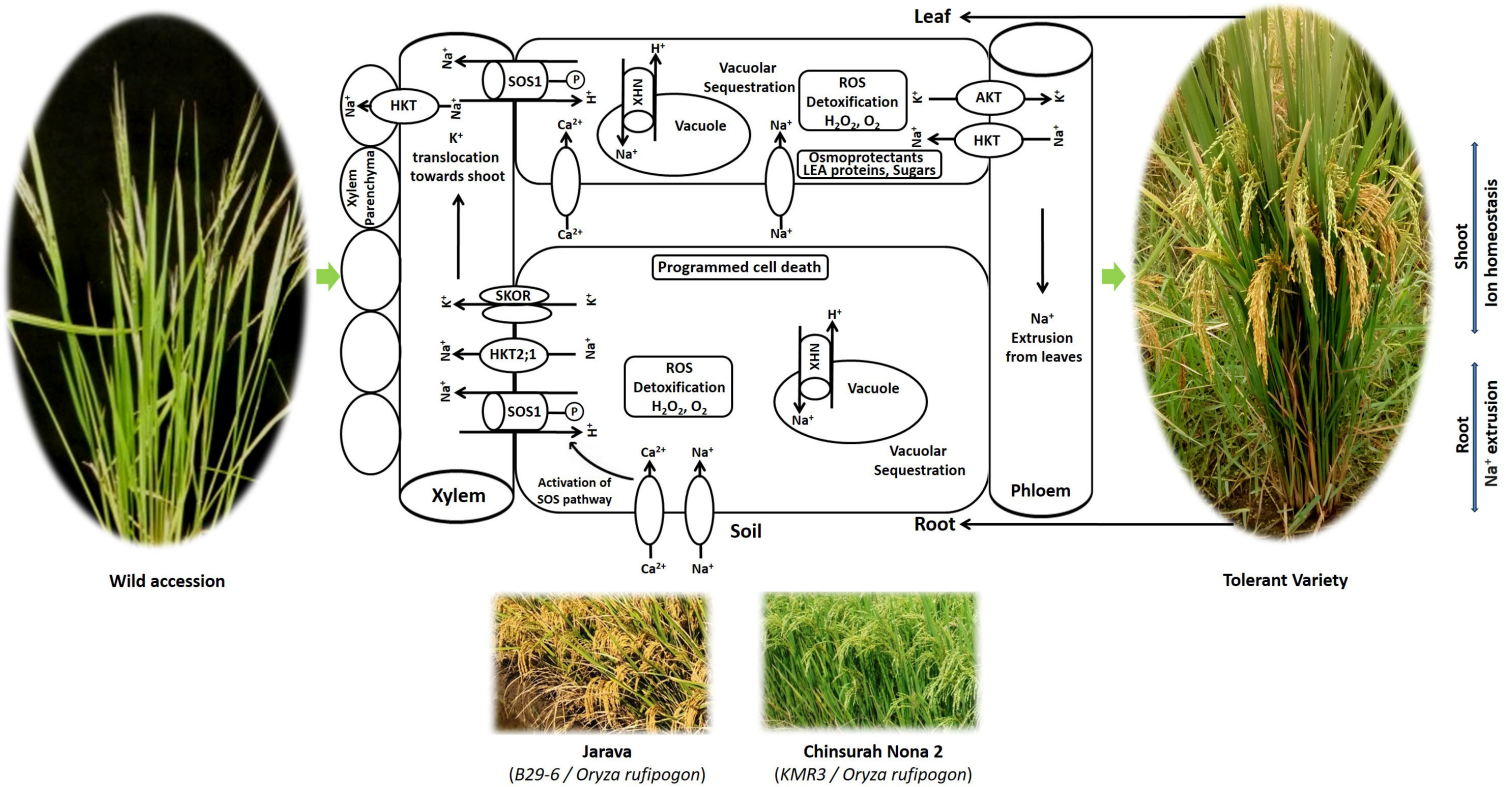
An overview of salt-tolerance mechanisms of rice under salinity stress.

Plants have evolved protective mechanisms at the cellular, organellar, and whole plant levels to recover from salinity stress. Rice cultivars exhibit genetic variation in their adaptive strategies against salinity (Khan et al., 2020). The crop has developed a series of adaptive mechanisms, including (i) osmotic adjustment, (ii) compartmentation and ion homeostasis, (iii) antioxidant defense, and (iv) programmed cell death (**Figure 3**).

### 4.1 Osmotic adjustment by accretion of osmolytes

Plants have less access to water and nutrients under osmotic stress. To save water and minimize transpiration, which causes Na^+^ ion inflow from the roots to the shoots, plants respond by closing their stomata (Flowers and Flowers, 2005). Osmotic stress builds up Na^+^ in the leaves and reduces growth (Afrasyab et al., 2010). Plants respond to salinity-induced osmotic stress by synthesizing osmoprotectants in the cytoplasm, such as quaternary amino acid derivatives (proline and glycine betaine), sugars (glucose, fructose, and trehalose), sugar alcohols (glycerol and methylated inositols), and late embryogenesis-abundant (LEA) proteins to maintain high cytosolic osmotic adjustment (Horie et al., 2012). The proline synthesis genes *OsP5CS1* and *OsP5CR* enhance proline biosynthesis and salt tolerance (Sripinyowanich et al., 2013). Glycine betaine, encoded by *OsCMO* and *OsBADH1*, accumulates in rice when exposed to high salt (Tang et al., 2014). *OsSWEET13* and *OsSWEET1* regulate sugar homeostasis in rice under saline conditions (Mathan et al., 2020). Salt and drought tolerance was considerably increased by the LEA genes *OsLEA3-2*, *OsLEA4*, *OsLEA5*, and *OsEm1* (Duan and Cai, 2012).

### 4.2 Compartmentation and ion homeostasis or tissue tolerance

Tolerant plants minimize the initial entry of Na^+^, restrict Na^+^ movement in the xylem, induce outflow of Na^+^ in the soil, enhance the absorption of K^+^, and regulate the Na^+^/K^+^ ratio to protect the leaves from ion toxicity (Munns and Tester, 2008). In rice, plasma membrane-based histidine kinase transporters (HKTs) modulate the accumulation of Na^+^ in the cytosol. They either absorb sodium from the soil solution or remove sodium from the xylem sap and load Na^+^ into the phloem sap to reduce sodium accumulation in leaves,when K^+^ is limited (Campbell et al., 2017). In the *Saltol* region of FL478, a Pokkali derivative and a sodium transporter gene, *OsHKT1;5*, mediate Na+ exclusion in rice (Hauser and Horie, 2010).

The capacity of a tissue to function normally while containing a high internal concentration of Na^+^ is known as tissue tolerance. It involves the sequestration of excessive Na^+^ from the cytoplasm into the vacuoles of non-functional older leaves and leaf sheaths, and enzymatic detoxification of reactive oxygen species. Selective uptake of Na^+^ into vacuoles is performed by four vacuolar Na^+^/H^+^ transporters (*OsNHX1*, *OsNHX2*, *OsNHX3*, and *OsNHX4*). Thus, plants maintain a high tissue K^+^/Na^+^ ratio, which prevents cytosolic Na^+^ toxicity (Wu et al., 2020). Excess Na^+^ is pumped out from the root xylem by a salt overly sensitive 1 (SOS1) transporter, the plasma membrane Na^+^/H^+^ antiporter, and *OsNHX1* and *OsSOS1* (Kumar et al., 2013) and *OsTPC1* (Kurusu et al., 2012), which contribute to ion homeostasis. Tissue tolerance also involves the biosynthesis of compatible solutes and the formation of enzymes, such as superoxide dismutase and catalase, which detoxify ROS and conserve cell size and turgor (Munns et al., 2016).

### 4.3 Defense against oxidate damage through detoxification of reactive oxygen species

Excessive reactive oxygen species (ROS) are formed in chloroplasts, peroxisomes, and mitochondria under salt stress. This causes oxidative damage to lipids, proteins in cell membranes, enzymes, and nucleic acids, and death in plants (Arif et al., 2020). Rice plants overproduce enzymatic antioxidants, *viz*., glutathione peroxidase, superoxide dismutase, catalase, and ascorbate peroxidase, and non-enzymatic antioxidants, such as proline, glycine betaine, glutathione, and polyphenols, to protect the cell against oxidative damage (Kim et al., 2017; Meng et al., 2018).

### 4.4 Programmed cell death

Plants adopt PCD to destroy excess or injured cells and prevent sodium influx into the shoots and roots. Reports have suggested that the upregulation of genes linked to PCD in rice increases salinity tolerance (Hoang et al., 2015). Cell death in rice roots during salinity stress was found to occur in a highly regulated manner (Liu et al., 2019).

Other tolerance mechanisms manifest through early vigorous growth to avoid salt toxicity and enhanced stomatal closure in rice (Kumar et al., 2013). The osmotic tolerance phase has not been much researched in rice compared to ionic stress. Tolerant rice accessions possess only a few of these mechanisms. Pooling the superior genes associated with adaptive mechanisms governing low Na^+^ uptake, high K^+^ uptake, Na^+^ sequestration, reduced transpiration, and synthesis of osmolytes to develop highly tolerant elite cultivars is urgently needed.

## 5 Overview of morphological, physiological, genetic, and molecular mechanisms underlying salt tolerance in wild rice

Wild species have evolved stronger morphological, physiological, genetic, and molecular adaptive mechanisms than cultivars have. They exhibit sodium secretion through special structures such as salt glands, improved ion homeostasis in roots and shoots, increased osmolyte biosynthesis, strong tissue tolerance, greater detoxification of ROS, and enhanced osmotic tolerance compared to cultivars. Several wild rice donors with increased tolerance to salinity have been, i.e., *O. coarctata* (Majee et al., 2004; Sengupta and Majumder, 2010; Garg et al., 2014), *O. punctata*, *O. officinalis* (Prusty et al., 2018), and *O. rufipogon* (Tian et al., 2011; Zhou et al., 2016).

Nakamura et al. (2002) reported higher seedling survival and high photosynthetic rates in *O. latifolia* and *O. rufipogon* up to 300 mM NaCl concentration than cultivated salt tolerant SR26-B and salt sensitive IR28. *O. coarctata*, earlier known as Porteresia coarctata, an allotetraploid wild rice halophyte with KKLL genome (2n = 4x = 48), is the only distant rice which could withstand extreme salinity (500 mM to 650 mM NaCl) as reported by Sengupta et al. (2008). It is a unique wild rice that grows in mangroves along coastal belts. It exhibits multiple defense mechanisms of tolerance and mainly adopts a salt excretion strategy to reduce the high salt load in photosynthetic tissues (Sengupta and Majumder, 2010), thereby retaining a high photosynthetic rate. It contains characteristic hairs known as trichomes on the upper surface of the leaves, through which sodium and chloride salts are excreted (Prusty et al., 2018). It deploys vacuolar Na^+^ compartmentation mediated by Na^+^ transporters (*OcNHX1*, *OcSOS1*, *OcHKT1;4*, and *OcHKT1;5*) to maintain a low cytosolic Na^+^/K^+^ ratio in the leaf mesophyll, even though it continues to grow in saline water. Despite high salinity, it sustains a low water content (Senthilkumar et al., 2005). Additionally, it possesses a greater Na^+^ and K^+^ retention capacity in shoots than in roots and eliminates ROS through enzymatic processes (Sengupta and Majumder, 2009).

A study conducted by Platten et al. (2013) using 103 *O. sativa* and 12 *O. glaberrima* accessions indicated the operation of ion homeostasis mechanisms in *O. glaberrima* through salt accumulation in leaves, which was independent of *OsHKT1;5*, as observed in *O. sativa*. Pushpalatha et al. (2013) evaluated 15 *O. nivara* and *O. rufipogon* introgression lines in the background of KMR3 and Swarna cultivars at various salinity levels (0 mM to 200 mM NaCl) during germination, vegetative, and reproductive growth stages and study revealed a Na^+^ exclusion mechanism and osmoprotection by proline synthesis in KMR3 ILs (K463 and K478), vacuolar sodium sequestration, and consistent chlorophyll content in Swarna ILs (S166, S3-1, S14, and S75) in response to salt stress.

Nishizawa et al. (2015), (Nishizawa et al., 2016) reported similar constant photosynthetic rates and sodium accumulation in *O. officinalis* and *O. latifolia* accessions. However, a relationship between stomatal conductance and net photosynthetic rate could not be demonstrated in *O. latifolia* compared to *O. officinalis*, indicating the possibility of the presence of a new set of salinity tolerance loci. *O. rufipogon* and *O. nivara* have been reported to possess a pool of genes that maintain K^+^ homeostasis, Na^+^ exclusion, and sodium compartmentalization (Ganeshan et al., 2016; Tian et al., 2017). Another wild relative, *O. australiensis* recorded proline accumulation, low Na^+^ content, and a low Na^+^/K^+^ ratio in the shoots and roots (Yichie et al., 2018).

Prusty et al. (2018) investigated 22 wild species in a hydroponic experiment along with four cultivated tolerant checks (Nona Bokra, Pokkali, and FL478) and two sensitive checks (IR 29 and IR 75862-206-2-8-3) under high saline stress (240 mM NaCl). Two wild species, *O. latifolia* and *O. alta*, survived up to 26 days to 33 days, and *O. coarctata* grew without deleterious effects. Interestingly, *O. eichingeri*, *O. minuta*, and *O. coarctata* accumulated high Na^+^ content in their roots and had low oxidative damage. Gene expression studies suggested the involvement of the *OsHKT1;4* transporter gene for mediating Na+ exclusion in leaves, but Na compartmentation occurred independently of the tonoplast-localized *OsNHX1* transporter gene. Shahzad et al. (2022) attributed the tolerance in wild rice genotypes of *O. alta* and *O. barthii*, to a greater tissue tolerance mechanism. Orthologous alleles of the stress-responsive *VOZ* gene from wild species, viz., *O. brachyantha*, *O. longistaminata*, and *O. nivara*, could act as potential donors for salinity stress improvement (Ganie et al., 2020). Nan et al. (2020) identified activation of *OrWRKY* genes in *O. rufipogon* under salt stress.

Nguyen (2022) investigated physiological traits governing salt tolerance in 18 Australian wild rice accessions of *O. australiensis*, *O. rufipogon*, and *O. meridionalis* under high salinity (200 mM NaCl) together with three cultivars, namely IR 29, salt sensitive and Pokkali, salt tolerant. *O. australiensis* accessions displayed high net photosynthesis, high relative water content, and low Na^+^ and Na^+^/K^+^ in the shoots and roots. Gene expression analysis revealed upregulation of proline synthesis genes *OsP5CS1* and *OsP5C2*, and downregulation of the proline degradation gene *OsProDH*. Thus, osmoregulation and ion homeostasis are the key tolerance mechanisms in *O. australiensis* accessions. Solis et al. (2022) demonstrated higher Na^+^ uptake and reduced Na^+^ effluxes in *O. alta*, *O. latifolia*, and *O. coarctata*. The expression of *NHX1* and *SOS1*/*NHX7* genes that govern tissue tolerance triggered by salt stress. Detailed information on the candidate genes and their mechanisms are listed in **Table 2**.

**Table 2:** List of salt tolerance adaptation mechanisms and the candidate genes in wild rice donors.

Wild *Oryza* donors	Candidate genes for salt tolerance	Salt adaptation strategy	Introgression lines/Cultivars developed	References
*O. coarctata*	*NHX1*, *VHA PsbR, MT2b, MT, MT2, MT3, L18a, L23a, PP, VPS2.1, IMT1, INO1, NACs, MYBs, WRKYs, OEC, MSP, CP47/PsbB, PsaE*, Rubisco activase, chloroplastic precursor of glutamine synthetase*, Hsp70*, cellulose synthase-like protein	Na^+^ sequestration, Salt exclusion through salt hairs, Unaltered carbon fixation and higher water retention, Higher synthesis of osmoprotectants, Higher ROS scavenging, Higher RUBISCO activation, cell wall synthesis and chaperone functions	IR56 ILs	Sengupta and Majumder (2009); Sengupta and Majumder (2010); Garg et al. (2014); Mishra et al. (2016); Prusty et al. (2018)
*O. rufipogon*	*OsGH3-2, OsGH3-8, CML15, GEM, LRP1, ABF2, RPK1, DST, HKT2;3, HKT1;5, BADH2, HsfC1B, MIPS1, MIPS2, MYB2, NHX1, NHX2, NHX3, P5CS1, P5CS2, PIP1, SIK1, SOS1*, and *SOS2*	Na^+^ retrieval from shoot, Higher ROS detoxification, Chlorophyll retention	Chinsurah Nona 2, Jarava, BRRI Dhan 55(AS996), Swarna ILs, KMR3 ILs	Tian et al. (2011); Nishizawa et al. (2015); Nishizawa et al. (2016); Mishra et al. (2016); Ganeshan et al. (2016); Wang et al. (2017); Prusty et al. (2018)
*O. latifolia*	*HKT1;4, HKT1;5, SOS1*	Na^+^ retrieval from shoot, Na^+^ exclusion, Na^+^ accumulation in mature leaves, Chlorophyll retention	–	Nishizawa et al. (2015); Nishizawa et al. (2016) *;* Prusty et al. (2018)
*O nivara*	*OsHTK1;1; OsHTK1;2 OsHTK1;3; OsHTK1;4 OsHTK1;5; OsHTK2;1 OsHTK2;3 OsHTK2;4*	Ion homeostasis	–	Mishra et al. (2016)
*O. alta*	*HKT1;5, SOS1*	Na^+^ retrieval from shoot, Na^+^ exclusion, Chlorophyll retention	–	Prusty et al. (2018)
*O. grandiglumis*	*HKT1;5, SOS1*	Na^+^ retrieval from shoot, Na^+^ exclusion	–	Prusty et al. (2018)
*O. officinalis*	–	Higher chlorophyll synthesis, photosystem not affected and higher water use efficiency	–	Nishizawa et al. (2016); Prusty et al. (2018)
*O. australiensis*	–	Leaf Na^+^ loading, High K/Na, Chlorophyll retention	–	Prusty et al. (2018); Yichie et al. (2018)
*O. australiensis*	*OsP5CS1* and *OsP5C2*	High net photosynthesis, high relative water content, high proline biosynthesis, low Na^+^ and Na^+^/K^+^	–	Nguyen (2022)
*O. glaberrima*	–	Na^+^ exclusion, low leaf Na^+^ concentration	–	Platten et al. (2013)

Source: modified from Solis et al. (2020).

Adaptive mechanisms may occur singly or in combination, depending on the species and growth stage of the crop. Different accessions of the same wild *Oryza* species could have a distinct genetic basis for phenotypic expression. With better knowledge of the mechanisms in wild species, appropriate breeding methods could be formulated to enhance yield under salt stress than what is currently obtained in studies involving *O. sativa* donors.

## 6 Approaches to improve salt tolerance in rice

Tolerance to salinity is a complex polygenic trait linked to several morphological and physiological traits, with huge environmental influence and poor heritability (Gregorio and Senadhira, 1993; Flowers, 2004). Based on an earlier standard hydroponic system of screening (Gregorio et al., 1997) and the modified Yoshida culture-based method (Singh et al., 2010), there have been several investigations in screening the germplasm of *O. sativa* subspecies *indica* and *japonica* (Negrao et al., 2011) to identify donors. In rice, three major strategies, namely conventional breeding, molecular breeding, and genetic engineering, were employed to generate superior salinity-tolerant cultivars.

### 6.1 Traditional breeding

Breeders have employed introduction, hybridization, pedigree selection, bulk method, modified bulk pedigree, recurrent selection, backcross method, and induced mutations to adapt to salt stress. Early breeding efforts have focused on improving locally domesticated landraces using pure line selection. Notable among them are Pokkali, Nona Bokra, Bhura Rata, and Kalarata. Globally, Pokkali is the most extensively utilized donor because it maintains a low shoot Na^+^/K^+^ ratio with tissue tolerance under high salinity. Unfortunately, linkage drag contribute to poor yield and grain quality, and the late maturity of landraces is often brought into new cultivars (Solis et al., 2020).

Approximately 101 salinity-tolerant rice cultivars developed using conventional breeding techniques have been developed worldwide (Singh et al., 2021). However, many of them are only moderately tolerant to salinity and not during all growth stages of the crop. The complex inheritance, pleiotropy, and high G × E interactions of salt-tolerance traits hinder traditional breeding efforts (Cohen and Leach, 2019). It is vital to breed rice varieties that can withstand high salt levels without compromising the yield. Under these conditions, the marker-assisted introgression approach shows more promise in the rapid development of tolerant cultivars and in lowering the risk of unwanted linkage drag with the negative traits of wild species and landraces. This has increased the need for the integration of molecular breeding techniques into the breeding process (Ismail et al., 2007; Thomson et al., 2010).

### 6.2 Molecular breeding

With the advent of DNA-based markers, approximately 1,000 QTLs for salt tolerance in rice have been mapped (Prakash et al., 2022), and several candidate genes for salinity tolerance have been identified (**Supplementary Table 1**). The genetic regions corresponding to these QTLs and genes have been implicated in various molecular and physiological processes. The major landmark in salinity tolerance breeding was the detection of a *Saltol* QTL for shoot K^+^/Na^+^ homeostasis on chromosome 1 (Gregorio, 1997) in an RIL designated as FL478 (IR66496-3R-78-1-1) derived from Pokkali/IR29. In the *Saltol* region, the *SKC1* gene of the Nona Bokra landrace controlling K^+^ concentration in shoots was identified and later cloned as *OsHKT1;5*, which encodes a plasma membrane Na^+^ transporter that regulates Na^+^ partitioning between roots and shoots (Ren et al., 2005).

*Saltol* QTL have been successfully transferred to many popular varieties through marker-aided back cross breeding (MABB) in India, Bangladesh, Russia, and Vietnam. (Padmavathi et al., 2023). The tightly linked markers within the *Saltol* QTL region (AP3206, RM8094, and RM3412), flanking markers, i.e., RM1287 and RM10694, RM493 and RM10793 enabled its successful transfer (Thomson et al., 2010) in the genetic background of BT7 (Linh et al., 2012), AS996 (Huyen et al., 2012), Bacthom 7 (Vu et al., 2012) in Vietnam, BRRI Dhan 49 (Hoque et al., 2015) in Bangladesh, Novator (Usatov et al., 2015) in Russia; ADT43 (Geetha et al., 2017), PB 1121 (Babu et al., 2017), Pusa Basmati 1 (Singh et al., 2018), Yukinko-mai (Rana et al., 2019), Pusa44 and Sarjoo 52 (Krishnamurthy et al., 2020), Pusa Basmati 1509 (Yadav et al., 2020), Aiswarya (Nair and Shylaraj, 2021) and Improved Samba Mahsuri (Rekha et al., 2022) in India. These MAS-derived varieties are already available to farmers for cultivation purposes.

Numerous QTLs for salinity tolerance in rice have been identified through biparental mapping populations. However, this approach may only partially reveal the genetic diversity of traits. A genome-wide association study (GWAS) facilitates the detection of a wide array of QTLs, thereby revealing a more extensive genetic diversity of the trait than bi-parental populations. Due to the higher recombination rate of the genome of natural genotypes, GWAS is employed for high resolution rapid mapping of genome-wide SNPs associated with morphological, physiological, photosynthetic and yield and its component traits under salinity such as K^+^/Na^+^ ratio, salt injury score, Na^+^ and K^+^ content of root and shoot, Na^+^ sheath: blade ratio, seedling length, fresh and dry weight of shoots and roots, chlorophyll and water content, number of panicles, filled grains and grain yield in seedling stage using hydroponics (Batayeva et al., 2018; Lekklar et al., 2019; Rohila et al., 2019; Zhang et al., 2020; Yuan et al., 2020; Le et al., 2021; Nayyeripasand et al., 2021; Kim and Suk-Man, 2023) and during the reproductive stage (Theerawitaya et al., 2020; Warraich et al., 2020; Chen et al., 2022).

The enrichment of MABB-derived salt-tolerant cultivars is constrained to bring major advancement, as it can only correct the deficiency of popular varieties, for example, salt sensitivity retaining the recurrent parent genome rather than creating highly heterotic salt-tolerant varieties. Wild rice has not been exploited much compared to cultivated rice. With the advent of next-generation sequencing (NGS) techniques, the available sequence information of genomes of seven wild species, viz., *O. rufipogon*, *O. nivara*, *O. barthii*, *O. glumaepatula*, *O. meridionalis*, *O. punctata*, and *L. perrieri* (Stein et al., 2018), provides opportunities to detect new genes and novel functional markers for incorporation into cultivars.

### 6.3 Genetic engineering

Genetic engineering is a promising approach for trait transfer to overcome hybridization barriers. This approach for increasing salinity tolerance centers on manipulating genes encoding the synthesis of compatible osmotica, antioxidants, sodium/potassium transport proteins, and transcription factors underlying salt tolerance mechanisms, focusing on cultivated rice (**Table 3**). Despite the improvement in transgenic rice possessing the reported genes produced under glasshouse conditions, they have hardly reached farmers’ fields for commercial cultivation (Kotula et al., 2020). Transgenic methods have focused only on altering individual genes and a single tolerance mechanism that hinders salinity tolerance improvement. Hence further research is needed to harness the potential of these wild sources.

**Table 3:** Genetically engineered rice varieties with increased salinity tolerance.

Transgene	Source of gene	Mechanism	Target rice variety	Reference
*OsPP1a*	Rice	Increased antioxidant enzymes (APX and SOD)	Rice	Liao et al. (2016)
*HsCBL8*	Wild barley	Proline accumulation and a reduced Na^+^ uptake	Zhonghua11	Guo et al. (2016)
*OsNHX1*	Pokkali rice	K^+^mediated osmoregulation	Binnatoa	Amin et al. (2016)
*PDH45*	Pea	Reduced Na^+^ accumulation and ROS	IR 64	Nath et al. (2015)
*PcINO1* and *PcIMT1*	*Porteresia coarctata*	Upregulated inositol metabolic pathway	IR 64	Mukherjee et al. (2019)
TPSP	*E. coli*	Enhanced K^+^/Na^+^ ratio, stomatal conductance, and photosynthetic efficiency	IR 64	Joshi et al. (2020)
*OsSOS1*	Rice	Na^+^ extrusion into apoplast	Vikas	Awaji et al. (2020)
*OsRF1*	Rice	Intensification of ABA signaling pathway	Dongjin	Kim et al. (2022)
*SiMYB19*	Foxtail Millet	Regulation of ABA synthesis and signal transduction.	–	Xu et al. (2022)
*miR5505*	Pokkali	–	–	Fan et al. (2022)

### 6.4 Genome editing

The technique permits editing of the target locus, knockout, and allele exchange in the genome, and culminating in the development of transgene-free edited plants. In rice, the CRISPR/Cas9 gene editing method has been effectively used to edit the *OsRR22* gene, which encodes a transcription factor that controls signaling and cytokinin metabolism in plants, thereby improving salt stress tolerance in rice (Zhang et al., 2019). The mutated salt-tolerant gene (*OsDST A*), which was developed using CRISPR/Cas9, has been reported to increase tolerance to salt stress by decreasing stomata and increasing leaf water retention in the MTU1010 rice variety (Santosh Kumar et al., 2020). In another study, CRISPR/Cas9 mediated mutagenesis of the BEARI transcription factor enhanced the tolerance to excessive salts by controlling ion transport (Teng et al., 2022). Improvement in salt tolerance with decreased salt build-up has been achieved by editing the *OsNAC3* gene in rice (Zhang et al., 2021). Mutants with CRISPR/Cas9-mediated *OsmiR535* knockout exhibit increased NaCl tolerance (Yue et al., 2020). The CRISPR/Cas9 mediated mutagenesis of the rice gene BG3, which promotes the transport of cytokinin hormones, revealed increased salinity tolerance (Yin et al., 2020). The CRISPR/Cas technique can be applied to investigate the wild rice gene pool and address challenges associated with linkage drag during the introgression of target wild genes into high-yielding backgrounds.

## 7 Deployment of wild rice relatives in breeding for salt tolerance

Wild rice species are treasure troves with various beneficial traits linked to yield, quality, and tolerance/resistance to abiotic and biotic stresses. Breeders often neglect the utilization of wild rice species for two main reasons. First, it is difficult to ensure gene flow from wild rice into the cultivated gene pool because of cross incompatibility, sterility, or non-viability in F_1_ or backcrosses, restricted genetic recombination between wild and elite genomes, and linkage drag from wild rice. However, these genetic complications could be resolved with MABB, ensuring precise gene introgression with selections to minimize unwanted linkage drag and backcrosses, as compared to the traditional approach (Warburton et al., 2017). Second, when wild rice is grown outside their native habitat, either it is poorly acclimated or the expression of beneficial alleles is concealed. Ultimately, the performance of the derived introgression lines (ILs) is inferior.

Novel genes from the AA genome containing wild species in the primary gene pool could be easily transferred into domesticated rice through the traditional back-cross method. However, distant crosses between *O. sativa* and genetically remote wild species of the secondary and tertiary gene pools are difficult to achieve due to cross incompatibility and embryo abortion and/or degeneration. However, embryo rescue techniques can overcome these hurdles by producing distant fertile interspecific hybrids (Jena, 2010). Intergeneric hybrids between *Porteresia coarctata*, distant rice relatives, and *O. sativa* were produced with limited success by adopting vegetative multiplication of rescued hybrid embryos (Jena, 1994). Salt-tolerant genes can also be incorporated from *O. porteresia* into *O. sativa* through bridge crossing with *O. australiensis* (Latha et al., 2004; Mammadov et al., 2018).

With the latest advancements in NGS technology and high-throughput phenotyping, the historical natural genetic variation for salt tolerance present in a panel of wild rice accessions can easily be captured following GWAS. This is a potential strategy for mapping salt tolerance genes/QTLs, particularly in wild rice, where cross-incompatibility complicates the generation of mapping populations. The use of genetically distant wild species in the development of improved cultivars with superior trait performance has been greatly facilitated by the identification of candidate genes, GWAS, and development of introgression lines through MABB (Singh et al., 2021; Dai et al., 2022).

Transgenic approaches using cloned genes from wild species offer a solution to the issue of direct introgression of wild rice genes. Most transgenic methods have focused on single genes and/or one type of salt-tolerance mechanism in cultivars. However, genetic engineering utilizing cloned genes governing antioxidants, osmolytes, and ion transporters, photosynthesis enhancement, and yield simultaneously could potentially lead to the creation of more resilient rice varieties capable of thriving under high-salinity conditions.

The double haploid technique (DH) is another potential method for generating new homozygous salt-tolerant lines from distant crosses involving wild species in a single generation from heterozygous parents, which would otherwise require several generations of selfing to achieve near homozygosity in conventional breeding.

Genome editing is an emerging strategy for speeding up the development of advanced breeding lines, wherein a specific site in a target wild gene is edited directly by insertion, deletion, or alteration of existing nucleotide/s in the genomic segment of commercial varieties developed through distant hybridization. It corrects the deficiency, as in the present case, of salt sensitivity of the cultivars by avoiding laborious conventional or MABB and the incorporation of large introgressed genomic regions of wild species. Speed breeding is another promising approach for utilizing wild progenitors in breeding that manipulates the photoperiod and temperature to rapidly generate multiple generations within a year. In scenarios where precise genome editing is not an option, speed breeding plays a pivotal role in accelerating generation advancement, enabling selection against undesirable traits inherited from wild species, and stabilizing the genetic background of newly developed cultivars.

Although extensive studies have not been carried out in rice using wild species for genome editing and transgenic approaches, a considerable amount of research has been conducted on QTL mapping and introgression of QTLs/candidate genes in agronomically superior cultures.

### 7.1 QTLs/genes in wild species

As the QTLs/genes from indigenous wild relatives cannot be utilized directly in breeding populations, pre-breeding strategies could be followed to identify and transfer wild rice genes into an intermediate breeding material that can easily be hybridized with modern elite varieties. Although wild rice is not as widely exploited as cultivated rice, it contains positive alleles, genes, and QTLs associated with salt tolerance. These effects have been documented using advanced backcross populations and introgression lines. Due to the large natural genetic diversity of wild species, QTLs associated with novel tolerance mechanisms could be prime candidates for improving salt tolerance (Stein et al., 2018). A few studies, as detailed below, have attempted to discover natural alleles governing salt tolerance in wild rice donors.

A collection of 87 ILs derived from Teqing/*O. rufipogon* was screened for salinity tolerance during seedling stage by Tian et al. (2011). They detected 15 QTLs, 13 containing *O. rufipogon* alleles that conferred higher tolerance in the Teqing background. These alleles enhanced relative root, shoot, and total dry weight at three loci (*qRRW10*, *qRSW10*, *and qRTW10*) on chromosome 10 in salt-tolerant ILs.

In a set of 285 ILs derived from 93-11/*O. rufipogon*, Wang et al. (2017) identified 10 QTLs for salt tolerance traits on chromosomes 1, 5, 7, 9 to 12 at seedling stage. They observed that *qST7* on chromosome 7 coincided with *qRRW7, qRSW7*, and *qRTW7* in *O. rufipogon* for salt tolerance reported by Tian et al. (2011). They also found that *qST10* on chromosome 10 shared a similar QTL hotspot as *qRRW10, qRSW10*, and *qRTW10* reported by Tian et al. (2011). Four candidate genes linked to salinity tolerance namely *LRP1* (*LOC*_*Os05g32070*), acetyltransferase (*LOC_Os05g31254*), GRAM domain containing protein (GEM, *LOC_Os10g34730*) and calmodulin-related calcium sensor protein (*OsCML15*, *LOC_Os05g31620*) were recorded in an *O. rufipogon* derived salt-tolerant IL 9L136. It is perceived that the accumulation of enzymatic antioxidants such as peroxidase, catalase and superoxide dismutase in 9L136 served as a probable antioxidant defense mechanism.

A RIL mapping population from a cross between salt sensitive cultivar, Ningjing16 and salt tolerant Dongxiang wild rice, *O. rufipogon* was used by Quan et al. (2018) to map 9 QTLs for salt tolerance at the seedling stage (*qST*) on chromosomes 1, 3, 4, 5, 6, 8, and 10. They reported that *qST6*, a major QTL influencing survival rate, accounted for 19.3% of the phenotypic variance and showed additive effects. They indicated protein kinases, MYB and zinc finger transcriptional factors and *SKC1, HKT1;5* transporters and *HAK6* as the potential genes within the QTL region. They hypothesized that ion homeostasis and kinase signalling pathways were the possible mechanisms of salt tolerance.

Wairich et al. (2021) conducted a research experiment to examine the effect of salinity stress on two different populations of interspecific introgression from *O. sativa* × *O. meridionalis* and *O. sativa* × *O. rufipogon* crosses. They identified three potential QTLs on chromosomes 1, 3, and 5 in *O. sativa* cv. Curinga *O. meridionalis* (Ng. acc. W2112) ILs, and 19 QTLs on chromosomes 1, 4, and 7 in *O. sativa* cv. Curinga *O. rufipogon* accession (IRGC 105491) population for various vegetation indices under salinity. The study demonstrated that introgression line (IL) CR47 of *O. sativa*/*O. rufipogon* cross had a tissue tolerance mechanism, while IL CM6, derived from *O. sativa*/*O. meridionalis*, had a higher Na^+^/K^+^ ratio in roots to cope with salinity.

Kumari et al. (2021) subjected back cross progenies derived from IR64 and *O. nivara* accession NKSWR 173 to screening against salinity stress. They genotyped a set of 74 BC_1_F_2_ families for the presence of seedling stage (*qSES1.1* and *qSES3.2*) and reproductive-stage salt-tolerant QTLs (*qSTY11.1*). Four backcrossed families displayed enhanced tolerance, as determined by phenotypic performance and QTL peak markers, during both stages of growth.

In another study employing backcross inbred lines (BILs) from the cross 9311 and an African wild rice, *O. longistaminata*, 18 QTLs conferring salt tolerance were found (Yuan et al., 2022), and one QTL each for salt injury score (*qSIS2*), the water content of seedlings under salt treatment (*qWCSST2*), and the relative water content of seedlings (*qRWCS2*) colocalized on chromosome 2. Sequence and expression analyses suggest that *MH02t0466900*, encoding cytochrome P450 86B1, may contribute to ion homeostasis.

In a study involving 117 DHs derived from F1s of Savitri and Pokkali, Samantaray et al. (2021) identified four candidate genes for salinity tolerance, namely *LOC_Os01g09550, LOC_Os01g09560*, *LOC_Os12g06560*, and *LOC_Os12g06570* during germination stage.

### 7.2 Wild rice derived salt-tolerant elite lines/cultivars

Elite rice lines with salinity tolerance have been successfully developed by harnessing the genetic variability in a limited number of wild rice species (Chatterjee et al., 2006; Tian et al., 2011; Ganeshan et al., 2016; Wang et al., 2017; Quan et al., 2018; Kumari et al., 2021; Wairich et al., 2021).

The wild species *O. rufipogon* (2n = 24, AA) is frequently used to breed salt-tolerant lines because of its close evolutionary proximity and high compatibility with O. sativa (2n = 24, AA) (Londo et al., 2006). The potential salt-tolerant introgression line, YIL 16, is a derivative of Teqing/*O. rufipogon* has been reported by Tian et al. (2011). It has three *O. rufipogon* genomic regions that hold *qRRW3* and *qSTS2*, which are responsible for the relative root dry weight and salt tolerance score, respectively. With its enhanced salt tolerance, YIL 16 could be directly utilized or suggested for breeding programs. Ganeshan et al. (2016) reported salinity tolerance genes in ILs derived from the crosses *O. sativa* with *O. rufipogon* or *O. nivara*.

Four candidate genes (*LOC_Os05g31254*, *LOC_Os05g31620*, *LOC_Os05g32070*, and *LOC_Os10g34730*) were identified in the QTL regions (*qRW10*, *qRSW10*, and *qRTW10*) of an introgression line (9L 136) according to a study carried out by Wang et al. (2017). This line was developed from a cross between a Chinese *O. rufipogon* accession and the *O. sativa* cultivar 93-1. The researchers hypothesized that the overexpression of these genes could improve salt tolerance in rice varieties. Furthermore, the potential of *O. rufipogon* QTLs to boost the antioxidant system in domesticated varieties could help them tolerate salt-induced oxidative stress through marker-assisted introgression.

Quan et al. (2018) employed an accession of wild rice, *O. rufipogon*, recovered DJ15, a salt-tolerant introgression line from O. rufipogon and O. sativa (Ningjing16) cross. Subsequently, six high-yielding salt-tolerant RILs developed between NIL DJ15/Koshihikari possessing both *qST1.2DJ15* and *qST6DJ15* were identified with an improved seed set.

**Figure 4:**
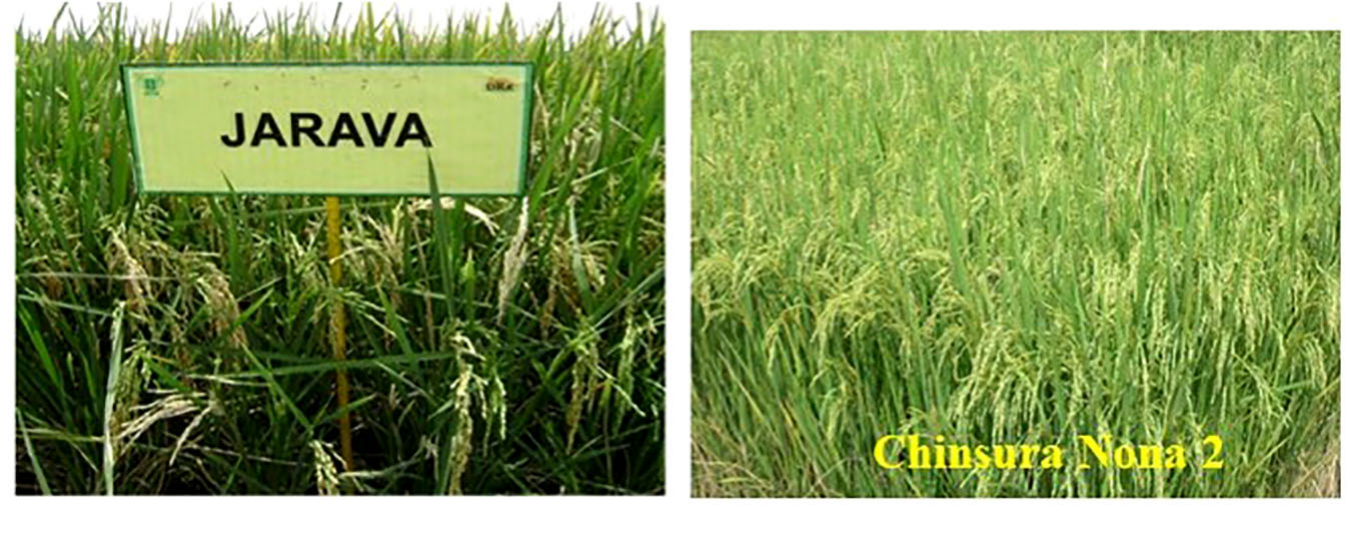
Wild rice derived salt tolerant rice cultivars released from ICAR-IIRR, Hyderabad, India.

Despite the availability of 22 potential wild species and two cultivated species (*O. sativa* and *O. glaberrima*), only two salt-tolerant varieties have been developed to date. Jarava and Chinsura Nona 2 were both developed using *O. rufipogon* donors. Jarava is a coastal saline-tolerant rice cultivar bred by ICAR-IIRR, Hyderabad, India, through distant hybridization between *O. rufipogon* and *O. sativa*. In 2005, it was released and notified by the Central Sub-Committee on Crop Standards, Notification and Release of Varieties for cultivation in West Bengal, Andaman & Nicobar, Puducherry states of India due to its superior yield under saline soils (Gazette of India notification No: S.O.1566 E dated 11-05-2005). It is a long-duration (143 days to 145 days) variety possessing short bold grains with 4.5 t/ha grain yield (**Figure 4**).

Another coastal saline tolerant cultivar, Chinsura Nona 2 (Gosaba 6), a medium duration (130 days to 135 days) variety with bold grains, originated from the KMR3 × *O. rufipogon* cross developed at ICAR-IIRR, Hyderabad in collaboration with Rice Research Station, Chinsurah, West Bengal, India (Thummala et al., 2022). It recorded 5.56 t/ha of grain yield under salinity; and was released and notified by the State Variety Release Committee (SVRC) for commercial cultivation in the West Bengal state of India (Gazette of India notification No S.O. 3220 E dated 5.9.2019). Thus, wild rice genes contributed immensely to the enhanced salt tolerance in the improved cultivars.

Employing genetic engineering, the MIPS coding gene from the most distant rice ancestor, *O. coarctata*, *PcINO1*, encoding L-myoinositol 1-phosphate synthase, was introgressed into the cultivated rice variety, Pusa Basmati-1, which displayed increased salt tolerance (Chatterjee et al., 2006).

## 8 Conclusions

Salinity, particularly during the reproductive stage, is a major abiotic stress factor that drastically reduces rice crop productivity. If not properly addressed, it poses a severe threat to global food security. Breeding for salinity tolerance is challenging due to its polygenic control, interaction with the environment, intricate physiological and metabolic processes, and growth stage. Despite the complexity, a moderate headway has been achieved with the development and release of approximately 101 salt-tolerant rice varieties using traditional global breeding methods.

The development of salt-tolerant cultivars has been hindered by the scarcity of genetic resources among domesticated cultivars. Therefore, future research should prioritize broadening the genetic base of modern cultivars by utilizing the salinity-adaptive genes present in unexplored wild species from secondary and tertiary gene pools. Prior to this endeavor, it is necessary to systematically explore and characterize various unexploited accessions of wild species for different salt stress defense mechanisms and identify genes for incorporation into breeding programs.

Obstacles associated with crossing and unwanted linkage drag are common during distant hybridization with wild species. This deficiency can be corrected by adopting the MABB method, which facilitates the accurate and accelerated introgression of target candidate genes/QTLs in popular cultivars. It is vital to reinforce pre-breeding programs that employ wild species in their breeding schemes worldwide. Continued research is needed to pinpoint more genomic resources in terms of salt-responsive QTLs and molecular markers and to profile the genes and their upstream regulatory regions in potential wild relatives. This will facilitate an in-depth understanding of the mechanisms that are effective in breeding tolerant varieties.

Exotic candidate genes have the potential to generate transgenic plants. The accurate pyramiding of genes responsible for different mechanisms of salt tolerance from divergent wild genetic sources into elite cultivars is needed for an hour to increase salt tolerance. Progress in transgenic and genome-editing techniques has paved the way for more possibilities to utilize and investigate valuable genes from wild rice to enhance salinity tolerance in cultivated rice.

The specific salt tolerance mechanisms in halophytes have yet to be fully elucidated. A comprehensive analysis of the process by which plants recognize salt stress and the crosstalk between different genes and pathways that are involved in regulating salt tolerance mechanisms is still needed. Concerted efforts are necessary to preserve both *ex situ* and *in situ* wild rice species for integration into salinity breeding programs.

## Author contributions

GP: Conceptualization, Writing – original draft, Writing – review & editing. UB: Writing – review & editing. KR: Writing – review & editing. DB: Writing – review & editing. MA: Data curation, Writing – review & editing. RKS: Writing – review & editing. RMS: Writing – review & editing.
