# Supplementary material for: Progress and prospects in harnessing wild relatives for genetic enhancement of salt tolerance in rice
Source: Front Plant Sci. 2024 Jan 31;14:1253726. doi: 10.3389/fpls.2023.1253726 (PMC10870985; doi:10.3389/fpls.2023.1253726)
Supplement: Supplementary file 1 [file Table_1.docx]

SUPPLEMENTARY TABLE 1 List of genes related to salinity tolerance in rice

| **Gene symbol** | **Gene name** | **Locus ID** | **Chr #** | **Position** | **Function** |
| --- | --- | --- | --- | --- | --- |
| CBSCLC1 | CYSTATHIONINE B-SYNTHASE DOMAIN CONTAINING CHLORIDE CHANNEL PROTEIN 1 | Os01g0876100 | 1 | 38017450..38022111 | CLC-type chloride channel homologue, Osmotic adjustment at high salinity, Adaptation to salinity (Os01t0876100-01) |
| MTI3A | METALLOTHIONEIN I-3A | Os01g0200700 | 1 | 5478545..5479749 | Metallothionein-like protein, Tolerance to salinity and heavy metal stresses, Abiotic stress response (Os01t0200700-01) |
| IF | INTERMEDIATE FILAMENT | Os01g0292700 | 1 | 10643287..10644848 | Intermediate filament, Cytoskeleton protein, Tolerance to salinity and heat stress (Os01t0292700-01) |
| GATA8 | GATA TRANSCRIPTION FACTOR 8 | Os01g0343300 | 1 | 13570702..13573119 | GATA transcription factor, Tolerance to abiotic stresses (salinity, drought, and ABA), Seed development (Os01t0343300-01) |
| SALT | SALT PROTEIN | Os01g0348900 | 1 | 13903285..13904626 | Jacalin-related mannose-binding lectin, Salinity tolerance, Resistance to M. oryzae (Os01t0348900-01) |
| GSTF11 | PHI GLUTATHIONE S-TRANSFERASE 11 | Os01g0353400 | 1 | 14167662..14168570 | Glutathione S-transferase, Tolerance to salinity and oxidative stress, ROS-scavenging activity (Os01t0353400-01) |
| PP2C8 | PROTEIN PHOSPHATASE 2C8 | Os01g0656200 | 1 | 26652966..26658299 | Protein phosphatase 2C family protein, Salinity tolerance in the seedling stage (Os01t0656200-01) |
| KAT1 | SHAKER POTASSIUM CHANNEL 1 | Os01g0756700 | 1 | 31761223..31763887 | Shaker potassium channel, Salinity stress tolerance, Ion homeostasis (Os01t0756700-01) |
| MPG1 | MANNOSE-1-PHOSPHATE GUANYL TRANSFERASE 1 | Os01g0847200 | 1 | 36395486..36398031 | Mannose-1-phosphate guanyl transferase, GDP-mannose pyrophosphorylase, Salinity stress tolerance, Ascorbic acid synthesis (Os01t0847200-01) |
| MYB48 | MYB TRANSCRIPTION FACTOR 48 | Os01g0975300 | 1 | 43096401..43097949 | MYB-related transcription factor, Drought and salinity tolerance (Os01t0975300-01) |
| CCR21 | CINNAMOYL-COA REDUCTASE 21 | Os02g0180700 | 2 | 4497115..4500124 | Cinnamoyl-CoA reductase 21, Response to biotic and abiotic stresses (M. grisea and Xoo infections, UV irradiation and high salinity) (Os02t0180700-01) |
| C2DP16 | C2 DOMAIN-CONTAINING PROTEIN 16 | Os02g0327000 | 2 | 13173784..13176182 | GTPase activating protein 1, Salinity stress tolerance, Abiotic stress response (Os02t0327000-01) |
| ASR1 | ABSCISIC ACID-STRESS-RIPENING-INDUCIBLE 1 PROTEIN | Os02g0543000 | 2 | 20165664..20166649 | Abscisic stress ripening protein 1. (Os02t0543000-01) |
| SOD2 | SUPEROXIDE DISMUTASE 2 | Os03g0219200 | 3 | 6271895..6275386 | CuZn superoxide dismutase, Response to salinity, drought, and oxidative stress (Os03t0219200-01) |
| GLYII-2 | GLYOXALASE II-2 | Os03g0332400 | 3 | 12263473..12266428 | Glyoxalase II (S-2-hydroxyacyl glutathione hydrolase), Salinity adaptation, Abiotic stress tolerance (Os03t0332400-01) |
| HAK21 | HIGH-AFFINITY POTASSIUM(K+) TRANSPORTER 21 | Os03g0576200 | 3 | 21060413..21064968 | High affinity K+ transporter (HAK), Maintenance of ion homeostasis, Salinity tolerance (Os03t0576200-01) |
| RGG1 | RICE G PROTEIN GAMMA SUBUNIT 1 | Os03g0635100 | 3 | 24252686..24256939 | Heterotrimeric G protein gamma subunit 1, Regulation of abiotic stresses, Salinity stress tolerance (Os03t0635100-01) |
| ZFP15 | ZINC FINGER PROTEIN 15 | Os03g0820400 | 3 | 34427704..34428391 | C2H2-type zinc finger protein, Positive regulation of of ABA catabolism, Salinity and drought tolerance, Seed germination (Os03t0820400-01) |
| MLP423 | MAJOR LATEX PROTEIN 423 | Os04g0465600 | 4 | 23278475..23279374 | Major latex protein, Bet v1 family protein, Positive regulation of drought and salinity tolerance (Os04t0465600-01) |
| FRO2 | FERRIC REDUCTASE 2 | Os04g0578600 | 4 | 29178862..29181665 | Similar to H0404F02.15 protein. (Os04t0578600-01) |
| C3H41 | ZINC FINGER CCCH DOMAIN-CONTAINING PROTEIN 41 | Os06g0318700 | 6 | 12352403..12358103 | Makorin RING finger protein, RING E3 ligase, Pattern formation and organogenesis during embryogenesis and post-embryogenesis, Negative regulation of salinity stress response (Os06t0318700-01) |
| SIK2 | STRESS INDUCED PROTEIN KINASE 2 | Os07g0186200 | 7 | 4593094..4595607 | S-Domain receptor like kinase-SIK2, Abiotic stress tolerance, Drought and salinity tolerance by modulating antioxidant capacity and eliminating H2O2 (Os07t0186200-01) |
| D-LDH2 | D-LACTATE DEHYDROGENASE 2 | Os07g0187200 | 7 | 4652793..4660423 | FAD linked oxidase, N-terminal domain containing protein. (Os07t0187200-01) |
| GATA23 | GATA TRANSCRIPTION FACTOR 23 | Os07g0615900 | 7 | 25372046..25376126 | GATA transcription factor, Multi-stress responsive transcription factor, Response to salinity and drought (Os07t0615900-01) |
| CCA1 | CIRCADIAN CLOCK ASSOCIATED 1 | Os08g0157600 | 8 | 3360865..3373384 | MYB transcription factor, Circadian clock, Tolerance to salinity, osmotic, and drought stresses (Os08t0157600-01) |
| YCHF1 | YCHF DOMAIN-CONTAINING PROTEIN 1 | Os08g0199300 | 8 | 5746738..5751829 | Ancient YchF-type P-loop NTPase, A member of the YchF subfamily, Unconventional G protein, Plant defense response, Salinity stress tolerance (Os08t0199300-01) |
| NAC63 | NAC DOMAIN-CONTAINING PROTEIN 63 | Os08g0436700 | 8 | 21218225..21220249 | NAC transcription factor, Tolerance to salinity stress (Os08t0436700-01) |
| ERF106 | ETHYLENE RESPONSE FACTOR 106 | Os08g0537900 | 8 | 26887813..26895496 | Similar to predicted protein. (Os08t0537900-01) |
| CCR17 | CINNAMOYL-COA REDUCTASE 17 | Os09g0127300 | 9 | 2083449..2086572 | Cinnamoyl-CoA reductase 17, Response to biotic and abiotic stresses (M. grisea and Xoo infections, UV irradiation and high salinity) (Os09t0127300-01) |
| RF1 | RING FINGER PROTEIN 1 | Os09g0478600 | 9 | 18351718..18352651 | RING-H2 type E3 ligase, Drought and salinity tolerance, Regulation of ABA signaling (Os09t0478600-01) |
| RNS4 | RIBONUCLEASE 4 | Os09g0537700 | 9 | 21155963..21157945 | S-like ribonuclease, Salinity tolerance, Abiotic stress response, Regulation of photomorphogenesis (Os09t0537700-02) |
| ACA8 | CA2+-ATPASE 8 | Os10g0418100 | 10 | 14681977..14685413 | Calcium-transporting ATPase, Salinity stress tolerance (Os10t0418100-01) |
| RLCK311 | RECEPTOR-LIKE CYTOPLASMIC KINASE 311 | Os11g0168600 | 11 | 3314083..3316292 | C-terminus of receptor-like cytoplasmic kinase, Salinity tolerance, Regulation of stomata response to stress (Os11t0168600-01) |
| MSL37 | MYB/SANT-LIKE 37 | Os11g0163500 | 11 | 3091820..3096321 | Trihelix transcription factor, Positive regulation of responses to salt stress, Salinity adaptation (Os11t0163500-01) |
| SADR1 | SALT, ABA AND DROUGHT STRESS-INDUCED RING FINGER PROTEIN 1 | Os11g0175500 | 11 | 3749274..3755192 | RING finger E3 ligase, Negative regulator in response to salinity (Os11t0175500-01) |
| U2AF65A | SPLICING FACTOR U2AF LARGE SUBUNIT A | Os11g0636900 | 11 | 25148722..25153451 | Similar to Splicing factor U2af large subunit A. (Os11t0636900-01) |
| YSS2 | YOUNG SEEDLING STRIPE 2 | Os12g0548300 | 12 | 22182885..22189908 | Nucleoside diphosphate kinase, Regulation of chloroplast development and chlorophyll biosynthesis, Abiotic stress response (ABA and salinity) (Os12t0548300-01) |
